# Characterizing the interface between wild ducks and poultry to evaluate the potential of transmission of avian pathogens

**DOI:** 10.1186/1476-072X-10-60

**Published:** 2011-11-15

**Authors:** Julien Cappelle, Nicolas Gaidet, Samuel A Iverson, John Y Takekawa, Scott H Newman, Bouba Fofana, Marius Gilbert

**Affiliations:** 1 CIRAD ES, UR Animal et Gestion intégrée des risques, Montpellier, France; 2 USGS Western Ecological Research Center, Vallejo, CA, USA; 3 Food and Agriculture Organisation, EMPRES Wildlife Unit, Infectious Disease Group Animal Health Service, Animal Production and Health Division, Rome, Italy; 4 Wetlands International, PO Box 471, 6700 AL, Wageningen, The Netherlands; 5 Direction Nationale des Eaux et Forêts du Mali, BP 275 Bamako, Mali; 6 Biological Control and Spatial Ecology, Université Libre de Bruxelles, av FD Roosevelt, 50, B-1050 Brussels, Belgium; 7 Fonds National de la Recherche Scientifiques, rue d'Egmont 5, B-1000 Brussels, Belgium

**Keywords:** Distribution modelling, Satellite Telemetry, Contact rate, Remote sensing, MODIS, GPS, Maxent

## Abstract

**Background:**

Characterizing the interface between wild and domestic animal populations is increasingly recognized as essential in the context of emerging infectious diseases (EIDs) that are transmitted by wildlife. More specifically, the spatial and temporal distribution of contact rates between wild and domestic hosts is a key parameter for modeling EIDs transmission dynamics. We integrated satellite telemetry, remote sensing and ground-based surveys to evaluate the spatio-temporal dynamics of indirect contacts between wild and domestic birds to estimate the risk that avian pathogens such as avian influenza and Newcastle viruses will be transmitted between wildlife to poultry. We monitored comb ducks (*Sarkidiornis melanotos melanotos*) with satellite transmitters for seven months in an extensive Afro-tropical wetland (the Inner Niger Delta) in Mali and characterise the spatial distribution of backyard poultry in villages. We modelled the spatial distribution of wild ducks using 250-meter spatial resolution and 8-days temporal resolution remotely-sensed environmental indicators based on a Maxent niche modelling method.

**Results:**

Our results show a strong seasonal variation in potential contact rate between wild ducks and poultry. We found that the exposure of poultry to wild birds was greatest at the end of the dry season and the beginning of the rainy season, when comb ducks disperse from natural water bodies to irrigated areas near villages.

**Conclusions:**

Our study provides at a local scale a quantitative evidence of the seasonal variability of contact rate between wild and domestic bird populations. It illustrates a GIS-based methodology for estimating epidemiological contact rates at the wildlife and livestock interface integrating high-resolution satellite telemetry and remote sensing data.

## Background

A large proportion (72%) of zoonotic emerging infectious diseases originate from wildlife, and most of the emerging disease hotspots are in tropical areas [1]. The wildlife-livestock interface is therefore of major interest because domestic animals are the most likely link between wildlife reservoirs and humans [2]. The wildlife-livestock interface is also of major interest for the study of economically important animal diseases like poultry diseases [3]. Research efforts on the wildlife-livestock interface are needed to better understand and control animal and zoonotic infectious diseases involving wildlife [4]. Characterizing the spatiotemporal interactions between wild and domestic animals is a key improvement of our knowledge of epidemiological dynamics [5]. The contact rate between wild and domestic hosts is one of the key parameters of the transmission of pathogens. Most of mathematical models of disease transmission have included β as a unique parameter that describes transmission [6]. β combines different effects of measurable biological parameters including the contact rate between hosts and the probability that contact events actually result in disease transmission [7]. Field assessments that accurately estimate the contact rate between hosts could improve estimates of β and the quality of epidemiological models, thereby supporting better predictions of disease dynamics.

Characterizing the contact between wild and domestic is now facilitated by recent technologies such as satellite telemetry and remote sensing. However, few studies have combined such advanced spatial tools to quantify the contact rates between wild and domestic hosts at high spatial and temporal resolution. Both direct and indirect contacts may lead to disease transmission. Direct contact requires that two hosts share the same space for a period of time that allows transmission of the pathogen. In contrast, indirect contact requires the survival of the pathogen in the environment for a period of time before infecting a new host. Indirect contact rates between wild and domestic animals are a function of the habitats they share during a period of time when the pathogen survives in the environment. Indirect contacts are a common way of transmission for major poultry diseases like avian influenza and Newcastle disease [8,9]. For free-living animals, animal observation [10] and telemetry [11] have been used to evaluate the direct contact rate between wild and domestic ungulates and canids. New technologies like proximity loggers provide new solutions to estimate direct contact rates between individuals [12]. In contrast, assessment of indirect contacts has been the subject of comparatively few studies.

Seasonal factors related to host populations or the environment are important drivers of disease dynamics [13], because they modify the transmission of infectious diseases and subsequent circulation [14]. Seasonality is a key element to consider in understanding space-time patterns of transmission of an endemic disease or to assess the risk of introduction of an exotic one [15,16]. Seasonal patterns have a strong influence on diseases circulating in wildlife, because key ecological traits like migrating or breeding are often timed to particular seasons of their annual cycle [17]. Thus, estimating seasonal variation of indirect contacts between hosts is critical to understanding the likelihood of disease transmission.

The main objective of this paper is to propose a method combining advanced spatial tools to characterize the wildlife-livestock interface. In order to estimate seasonal variation of indirect contact rates, we combined satellite tracking and remote sensing data to develop an indicator of spatiotemporal dynamics between wild ducks and domestic poultry in a tropical wetland. GPS satellite tracking provides numerous accurate locations for estimating contact rates of a limited number of individuals since it is very costly. The method we propose can be used at a local scale with a limited number of transmitters. However its results should not been extrapolated to other areas unless a consequent number of satellite transmitters is deployed.

We applied our method to assess the potential indirect contacts between comb ducks (*Sarkidiornis melanotos melanotos) *and poultry in the Inner Niger Delta (IND) in Mali. Both comb ducks and chickens have been tested positive for avian influenza viruses during the dry season in 2008 [18,19]. We ran our analysis every 8 days for successive periods during the study (a total of 15 consecutive 8-day periods from February to July 2007). For each period, our analysis included: i) estimating the spatial distribution of chickens based on investigations in villages, ii) modeling the spatial distribution of wild birds by combining satellite telemetry data of comb ducks and remotely sensed environmental indicators, and iii) identifying areas of potential indirect contact between chickens and wild birds. Concurrently, we compared the distribution of comb ducks and natural ponds to understand their use of the different types of habitat available in the area (mainly natural ponds, flooded plains and irrigated areas). Comb ducks are mostly using natural ponds as roosting sites during the day [20]. After these natural ponds dry out during the dry season, we expected the comb ducks to switch to other habitats, including irrigated areas in the vicinity of villages. This could increase the potential contacts between wild and domestic birds.

## Results

### Distribution of Poultry and Wild Birds

#### Distribution of poultry

Results from our investigations were consistent across all 21 villages investigated in the area and provided three main points: first, most poultry are chickens and they are kept in different households in the village during the night but are free-ranging together during the day; second, they use habitats within a 500 meters around villages during the day; and third, chicken dispersal pattern is consistent across the dry and the wet seasons. Thus, we considered a village as a relatively stable epidemiological unit for chickens. The spatial distribution of chickens incorporated the extent of the village at night but increased to include a 500-meter buffer during the day (Additional file 1 figure S1). We used the same spatial distribution for all 8-day periods.

#### Distribution of wild birds

**Figure 1:**
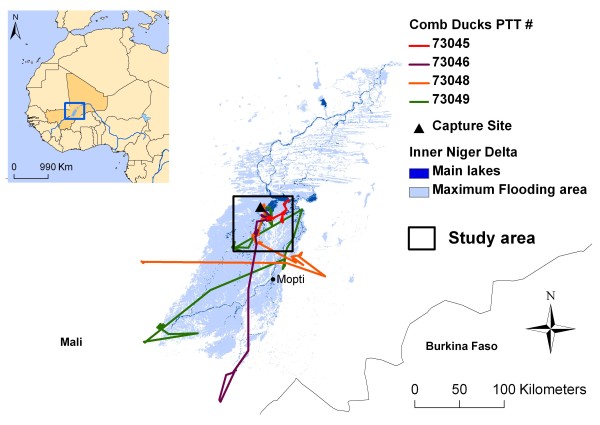
**Map of the Inner Niger Delta in West Africa and movements of four comb ducks tracked with satellite transmitters**. Marked ducks were tracked for up to 191 days and provided 5, 200 locations or an average 9 locations per day per transmitter.

The four birds marked with satellite transmitters were tracked during 191 days between 14 February during the dry season and 23 August during the rainy season. We obtained 5, 200 GPS locations, *i.e*. an average of 9 locations per day per bird (Table 1). Birds stayed in a relatively small area (70 km × 55 km) during the dry season and into the beginning of the rainy season (Figure 1). They made extensive movements during the middle of the rainy season to reach breeding areas outside of the Inner Niger Delta (IND) (Figure 1).

**Table 1:** Details of the GPS data sent by the four satellite transmitters attached on comb Ducks

Ptt ID	Duration (days)	No. locations	Max. distance from origin (km)
73045	79	810	30
73046	142	1541	223
73048	164	1451	146
73049	192	1398	200

**Figure 2:**
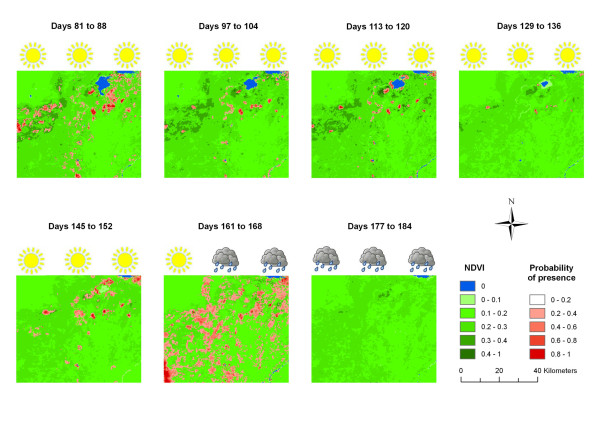
**Predicted spatial distribution of the comb ducks in the study area**. This figure shows a time series of maps of the probability of presence of the comb ducks in the study area for different 8-day periods. The probability of presence was estimated by the Maxent model run for each 8-day period with five remotely sensed indicators and the satellite tracking data of the comb ducks in the study area. After the beginning of the rainy season, all the birds have left the study area to reach their breeding grounds, explaining why no suitable area is predicted by the model for the last 8-day period (Days 177 to 184). The sun icons indicate the dry season while the rainy cloud icons indicate the rainy season.

The model of the spatial distribution of the comb ducks in the study area fits the GPS data well, and the training area under the curve (AUC) ranged from 0.820 to 0.998 (mean = 0.95, sd = 0.05) for the 15 successive 8-day periods. The indicators that contributed most to the fit of the model were distance to flooded vegetation with a mean contribution of 63% (sd = 16%) and NDVI with a mean contribution of 23% (sd = 17%). The probability maps indicated two different gridcell spatial patterns depending on the season (Figure 2). The number of cells identified as suitable by the model decreased during the dry season, but it increased toward the end of the dry season into the beginning of the rainy season (day 145-177) before the birds dispersed from the study area.

### Potential indirect contact between poultry and wild birds

**Figure 3:**
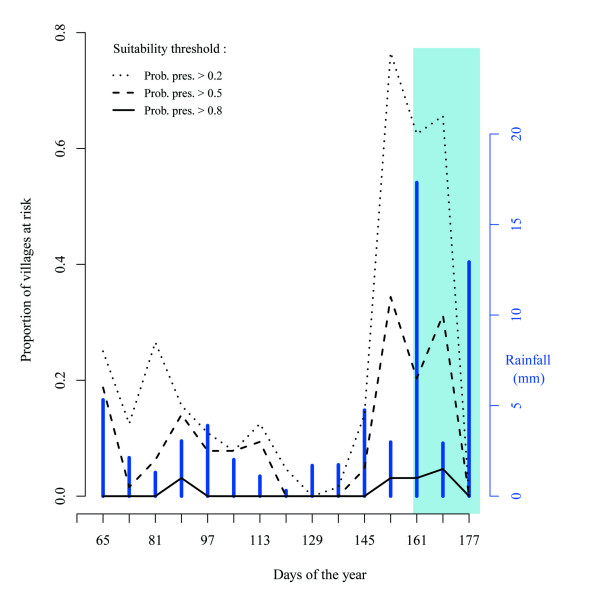
**Proportion of villages in potential contact with comb ducks**. This proportion is given for three different values (lines) of a suitability threshold. This threshold is the value of the predicted probability of presence above which a cell is considered as suitable for the comb ducks by the distribution model. The bars indicate the rainfall for the 8-day period. The light blue background indicates the rainy season.

Field measurements revealed that ponds and rivers in the IND were composed of freshwater with a pH close to 7 that ranged from 25°C to 35°C. Depending on the season, the survival of most strains of AIV and NDV in the environment was likely to be longer than a few days [8,21-24]. Although chickens only used the water bodies during the day, they could be infected by comb ducks that used those same habitats during the night. We recorded an indirect contact when a suitable model cell was in the chickens dispersal range (i.e. within 500 meters of a village). To quantify potential contacts between chickens and comb ducks, we calculated the number of villages in the area modelled as suitable for comb ducks for each 8-day period (Figure 3). The proportion of villages of the study area in potential contact with comb ducks decreases during the dry season (Figure 3). However, we observed. a period with increased potential contacts at the end of the dry season into the beginning of the rainy season (Figure 3).

### Correspondence between wild birds and natural ponds

**Figure 4:**
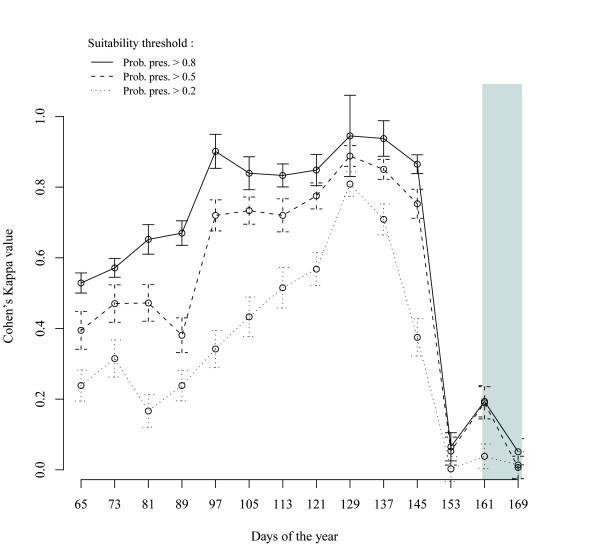
**Correspondence between areas predicted as suitable for comb ducks and natural ponds in the study area**. The correspondence is estimated by the Cohen's kappa value for three different values of a suitability threshold. This threshold is the value of the predicted probability of presence above which a cell is considered as suitable for the comb ducks by the distribution model. The light grey background indicates the rainy season.

The overlay correspondence between wild birds and natural ponds distributions varied temporally with the suitability threshold (Figure 4). Moderate (0.4 < kappa ≤ 0.6) to high agreement (kappa > 0.8) calculated for different thresholds indicated that natural ponds were suitable habitats for comb ducks. For all suitability thresholds (results for 0.2, 0.5 and 0.8 are displayed in Figure 4), agreement between suitable cells and natural ponds increased until the end of the dry season indicating that the birds were increasingly using natural ponds as the dry season progressed. Conversely, agreement was very low (kappa ≤ 0.2) at the end of the dry season into the beginning of the rainy season before birds left the study area.

The two main results are therefore that, first comb ducks are increasingly using natural ponds during the dry season which may be due to the drying out of other types of habitats like flooding plains, and second comb ducks switched to other type of habitats than natural pond at the end of the dry season. They could use irrigated areas or other artificial wetlands after the drying out of the natural ponds.

## Conclusions

Our results showed that satellite telemetry and remote sensing data may be combined to model indicators of key epidemiological parameters and their temporal variability with high spatial and temporal resolution. We were able to quantify seasonal variation in locations and timing of potential indirect contacts between wild ducks and chickens. We identified a critical period at the beginning of the rainy season that may have the highest potential for transmission and spread of pathogens in the IND due to regional movement. Our method can therefore be implemented at a local scale to assess the potential contacts at the wildlife-livestock interface in remote tropical areas.

The potential for contacts between comb ducks and chickens during the dry season may be explained by environmental dynamics. We observed that correspondence between predicted suitable cells for comb ducks and natural ponds increased during the dry season indicating that wild birds were likely increasingly using natural ponds. Dessication of these natural ponds in the vicinity of villages explains lower predicted contacts with chickens during the dry season. At the end of the dry season and the beginning of the rainy season, the agreement between predicted habitat used by comb ducks and natural ponds is very low (kappa < 0.2) indicating that the birds stopped using these habitats when they dried out. Wild birds moved to smaller ponds or irrigated areas during this period, increasing potential contacts with chickens.

The potential spread of pathogens by comb ducks is related to drivers leading to regional movements of wild birds at the beginning of the rainy season. Two main factors influenced the movement behaviour of comb ducks. First, refilling of many water bodies with seasonal rainfall led to the emergence of areas rich in food resources surrounding the IND [25]. Second, the breeding behaviour of the birds influenced their movements. Comb ducks, as many other tropical waterbirds species, breed mainly during the rainy season [20]. Their breeding sites, remote water bodies or flooded forests, are the only favourable habitats during the rainy season. The use of favourable habitats for both feeding and breeding during the rainy season led to regional movements that increased the potential spread of avian-borne pathogens by wild birds.

Validation and extrapolation of our results are limited by the type of data and the relatively small sample size used in our study. No validation (e.g cross-validation) method was convenient due to the spatial auto-correlation of the GPS data. We only showed the training AUC automatically calculated by Maxent. But a careful interpretation of these good scores of AUC (mean = 0.95, sd = 0.05) is required. First, AUC can be criticized for its reliability to accurately assess the performance of niche models [26,27]. And second, the spatial-autocorrelation of our data likely leads to an overestimation of the performance of the model. The absoltute value of potential contact rate estimated in this study would be hard to generalize to other species or other ecosystems due to the limited number of individuals from a single species we could monitored. Although we obtained numerous detailed GPS locations on four individuals, these individuals may have only represented movements of only one sub-population as the four tagged comb ducks went to the same pond in the study area. However, a field observation a year after the release of the tagged birds showed this pond was a major roosting for comb ducks and white-faced whistling ducks. One would expect birds from different family groups to congregate on this pond with other groups. Furthermore, the behaviour of the tagged comb ducks was in compliance with what is known about their ecology and what local hunters reported during our field work: they congregated on the remnant water bodies during the dry season and performed a regional movement to reach different breeding grounds during the rainy season [20,28]. That is why we believe these results can be extrapolated to other wild ducks in the study area. However, we would not extrapolate our results to other African wetlands without deploying more satellite transmitters in these areas. Studies with more transmitters would be more informative but their cost could be a major limitation. We believe that our study with few transmitters is interesting to locally assess the potential contacts between wild birds and chickens, and can be easily implemented, even in remote areas [29].

Contact rate between hosts, being either direct or indirect, is one of the main parameters of the transmission dynamics of infectious diseases. However several other parameters may modulate the probability for a contact to produce an effective transmission of pathogen. First, transmission is likely to be influenced by the density of hosts [13]. Evidence of density-dependent transmission of avian influenza has been shown for wild birds [30,31]. Second, transmission depends on the probability that the contact occurs between a susceptible and an infected hosts. Consequently, the proportion of infected hosts in the population and the level of population immunity are crucial parameters [17]. Finally, the duration of persistence of the pathogen in the environment will control the probability of transmission. This is particularly true for water-borne transmitted viruses like AIV for which temperature, pH, and salinity may reduce the duration of survival and infectivity of the virus in the environment [8,21,22,24].

Therefore, one has to consider the possibility that the period with the greatest contact rate between wild and domestic hosts may not be a period of maximum transmission.

In classical SIR models, transmission parameters are usually assembled in the parameter β [6]. Different components of the β could be considered separately, like contact rate and transmission rate. Estimating and modelling these two distinct components of the β separately should provide a better understanding of the transmission dynamics of infectious diseases. It would also allow the use of a direct measure of these parameters rather than estimating a global β, which is usually the case [32]. Our main results, the proportion of villages in potential contact with comb ducks, may be used to refine a seasonal forcing of the contact rate. In a SIR model with a density-dependent transmission, the force of infection λ would be expressed as:

λ(t)=p(t)×β.I/N

where p(t) is the proportion of villages in potential contact with comb ducks as a function of time, β is the probability that a domestic bird is infected with AIV following contact with a wild bird, I is the number of infected wild birds, and N is the size of the wild bird population [7,33]. Thus, our method could potentially be integrated into epidemiological models aiming to take into account the dynamic of contact rates between hosts. It would improve the efficiency of these models when contact rates are explicit parameters of the models [34]. Finally, our approach could be used to identify the villages with an increased risk of indirect contact with wild ducks. This would allow implementing risk-based surveillance in areas by targeting the villages with the highest risk of contact.

## Methods

### The Inner Niger Delta

The Inner Niger Delta (IND) in Mali is the largest continental wetland in West Africa and the second largest in Africa. Stretching over 41, 195 km^2^ in the midst of the Sahelian zone, this low elevation floodplain area includes a number of seasonally inundated lakes, ponds and river channels. Over one million people live in the area with their domestic animals. The IND is a key West African wetland and supports many wild birds species including up to one million migrating Palearctic ducks during the northern winter, 100, 000 Afro-tropical ducks, and 300, 000 waders of various origins [35]. The ecology of the area is mainly driven by the flood level, which itself depends on rainfall in the region [36]. After the rainy season (June to September) the area is flooded for several months (September to December), decreasing in water level in the following months when aggregations of waterbirds are found on the remnant water bodies (lakes and natural ponds) that are also used by people and chickens. The mixing of wild waterbirds and chickens provides favourable conditions for transmission of avian-borne pathogens like avian influenza viruses (AIV) or Newcastle disease viruses (NDV).

### Distribution of Poultry and Wild Birds

#### Distribution of Poultry

To document the distribution of poultry in the IND, we conducted investigations in 18 of the 64 villages in the area and collected information about the maximal distance of scavenging by poultry around villages. We developed a questionnaire comprising several questions about the poultry populations, husbandry and movements among the villages. The questionnaire was used in all villages investigated and the same procedure was applied in all villages involving each time a meeting with the village leader.

#### Wild birds Satellite telemetry data

On 17 February 2007, we deployed 30g solar-powered Platform Terminal Transmitters (PTTs; Microwave Telemetry Inc., Columbia, MD, USA) on four comb ducks (*Sarkidiornis melanotos*) at Barnajee (15.22°N, 4.31°W) in the IND (Figure 1). We selected the comb duck for its potential to spread pathogens regionally, in particular for AIV for which comb ducks had been tested positive in the Inner Niger Delta in 2006 [37]. It is a large African duck known to perform extensive intra-African movements, including trans-equatorial migration [20], and it breeds during the rainy season in sub-Saharan Africa. The PTT was < 3% of the bird's body mass [19], and we used an attachment technique [18] similar to the one described by Miller *et al*. [38] which proved successful in North America for tracking northern pintail (*Anas acuta*) during migration. Location data were uploaded via the Argos Data Collection and Location System (CLS, Toulouse, France) to receivers aboard polar-orbiting weather satellites. In order to conserve battery power, the PTTs were programmed to transmit data in a duty cycle of 6 hours every 2 days (48 h). The PTTs logged GPS locations every two hours when they had sufficient stored power from the solar panel with a mean accuracy estimated at ± 18.5 m. We only considered locations transmitted at least 15 days after the release of the birds, in order to discard any aberrant movements associated with capture, handling and harness attachment [39].

#### Environmental data

The IND is a remote area with a rapid environmental dynamic due to the seasonal flooding. In order to capture the space-time dynamics of environmental conditions, we used remotely sensed indicators from the Moderate Resolution Imaging Spectroradiometer (MODIS) sensor which provides a good trade-off between spatial (250 m or 500 m cells depending on bands) and temporal resolution (8 days). Different indicators have been developed to remotely monitor water bodies, whether open water or flooded areas [40]. We selected one indicator of vegetation and one indicator of water, because they are the two main environmental factors most likely to be related to the distribution of wild waterbirds. We used the Normalised Difference Vegetation Index (NDVI) [41], the most commonly used vegetation indicator, to assess vegetation. We used the Modified Normalised Difference Water Index (MNDWI) [42], found to be one of the most efficient water indicators, to monitor temporary ponds over large areas of arid lands [43]. These two indicators were available with a 250 m spatial resolution and an 8-day temporal resolution. We also created vegetation and water indicators to quantify their dynamics. These two other indicators were differences between the value of the NDVI or the MNDWI for the 8-day period considered compared with the value of the same indicator 24 days before. Finally, we used a fifth indicator, the distance to flooded vegetation, because we expected that the spatial distribution of wild waterfowl would be related to ponds with flooded vegetation, and there would likely be strong interaction between the NDVI and the MNDWI. High values of NDVI could be related to favourable habitat for waterbirds when associated with high values of MNDWI, indicating flooded vegetation used by waterbirds. On the other hand, high values of NDVI could also be related with non-favourable habitat when associated with low values of MNDWI, indicating terrestrial vegetation not used by waterbirds. This fifth indicator was calculated as the distance to the closest cell identified as flooded vegetation. A cell was considered to contain flooded vegetation when its NDVI value was greater than *v *= 0.2 and its MNDWI value was greater than *w *= -0.3 (see Additional file 1 for the determination of v and w).

#### Distribution modelling of wild birds

We used the maximum entropy (Maxent version 3.3.2) method developed by Philips *et al *[44] and adapted for ecological applications by Elith *et al *[45] to determine suitable habitats for comb ducks in the study area based on our environmental indicators and satellite tracking data. Maxent is a machine-learning method for making predictions or inferences from incomplete information. It was selected, because it is one of the most accurate methods for predicting species distribution [46], it is well adapted to satellite tracking studies providing presence-only data, and it can be run with limited training data. For all 15 consecutive 8-day periods, we ran the model to predict the probability of presence of comb ducks in the study area using 500 iterations of the Maxent sequential-update algorithm. Because of the auto-correlation between the GPS locations, no cross-validation was undertaken. Any subset of GPS points for training or testing the model would be very similar to the full dataset, leading to an overestimation of the model performance. We trained the model with all locations in the study area for all birds and the five environmental variables corresponding to an 8-day period. The model provided us with a time series of probability maps where comb ducks were present in the study area.

### Indirect contact between poultry and wild birds

An indirect contact may be defined when two hosts share the same space during a period of time corresponding to the survival of the pathogen in the environment. The survival in water of both AIVs and NDVs depends on temperature, pH and salinity [8,21,22,24]. To determine the potential of indirect contact between poultry and wild birds for these two pathogens, we considered for each 8-day period the spatial distribution of both poultry and wild birds, the timing of poultry and wild birds in these areas, and the estimated survival time of the pathogens in the environment depending on temperature, pH, and salinity.

### Correspondence between wild birds and natural pond distributions

We compared the spatial distribution of comb ducks with the spatial distribution of natural ponds to understand how comb ducks use the different habitats available in the study areas. Three main habitats are available for comb ducks, natural ponds, flooded plains, and artificial wetlands like irrigated areas. Natural ponds are well delimated water bodies (as opposed to flood plains) that are naturally flooded by rainfall or by the global flooding of the Inner Niger Delta(as opposed to water bodies flooded by men like irrigated areas). These different habitats have different dynamics that may influence the spatial distribution of comb ducks. We focused on the correspondence between wild birds spatial distribution and natural pond distribution because natural ponds can be easily spatially delimited and because we expected natural ponds to be the main habitat used by comb ducks. We did not expect perfect concurrence of comb duck spatial distribution and natural pond spatial distribution, because comb ducks also use other habitats like flooded plains or irrigated areas. Based on observations conducted during a field mission in March 2008 and on Google Earth™ images, we determined the location of the main natural ponds in the study area (Additional file 1 figure S1). We calculated a Cohen-Kappa coefficient [47] to evaluate the agreement between the probability of presence of comb ducks predicted by the model and the spatial distribution of the natural ponds. We used a sample of 200 cells of our study area to measures the agreement between the predicted distribution of the comb ducks (characterized by cells predicted as suitable by the model) and the distribution of the natural ponds (characterized by cells in which were observed natural ponds). For each 8-day period and for different suitability threshold, we randomly sampled 100 cells with a probability greater than the suitability threshold (suitable cells), and 100 cells with a probability lower than the suitability threshold (unsuitable cells). The suitability threshold is the value of the probability of presence predicted by the model above which a cell is considered suitable for comb ducks. This suitability threshold ranges from 0 to 1. When less than 100 suitable cells were available for analyses, we used all available suitable cells and an equivalent number of unsuitable cells.

A high agreement would indicate that comb ducks are using natural ponds as their main habitat. Lower agreement would indicate that comb ducks are using other types of habitats, like flooded plains or irrigated areas.

## Competing interests

The authors declare that they have no competing interests.

## Authors' contributions

JC and MG conceived and ran the spatial analysis. NG, SI, JT and SN planned the satellite telemetry study. JC, NG, SI and BF collected the data on the field. JC, NG, JT, SN and MG wrote and edited the paper. All authors have read and approved the final manuscript

## Supplementary Material

Additional file 1 **Construction of the fifth indicator and additional figures**. This file explains how was built the fifth environmental indicator used as an explanatory variable in our distribution model. We describe the two steps and the optimisation process leading to the variable called 'distance to flooded vegetation areas suitable for wild birds'. The files also contains figure S1 showing the 64 villages and the natural ponds and lakes included in the study area, and figure S2 showing typical result of the optimisation process used to build the fifth environmental indicator. Click here for file
