# Supplementary material for: Characterizing the interface between wild ducks and poultry to evaluate the potential of transmission of avian pathogens
Source: Int J Health Geogr. 2011 Nov 15;10:60. doi: 10.1186/1476-072X-10-60 (PMC3280937; doi:10.1186/1476-072X-10-60)

**Additional file 1**

**Construction of the fifth indicator: distance to flooded vegetation**

The fifth indicator was built in two steps. First (i) we identified all cells with a NDVI value superior to a threshold *v* and a MNDWI superior to another threshold *w*. These two thresholds were determined successively by minimising two parameters: the number of cells identified as flooded vegetation, and the mean distance of GPS points to cells identified as flooded vegetation. The minimum optimum calculated for all 8-day periods tested were used as *v* and *w* to consider all flooded vegetation areas during the study period. Second (ii), for all 8-day periods we calculated for each cell in the study area the distance to the closest cell identified as flooded vegetation, we thus obtained our fifth indicator: distance to flooded vegetation areas suitable for wild birds.

Based on our optimisation process, we determined a value of 0.2 for *v* and -0.3 for *w*. A typical result for this optimisation process is shown by Figure S2.

**Additional Figures**

**Figure S1. Map showing the 64 villages and the natural ponds and lakes included in the study area.**


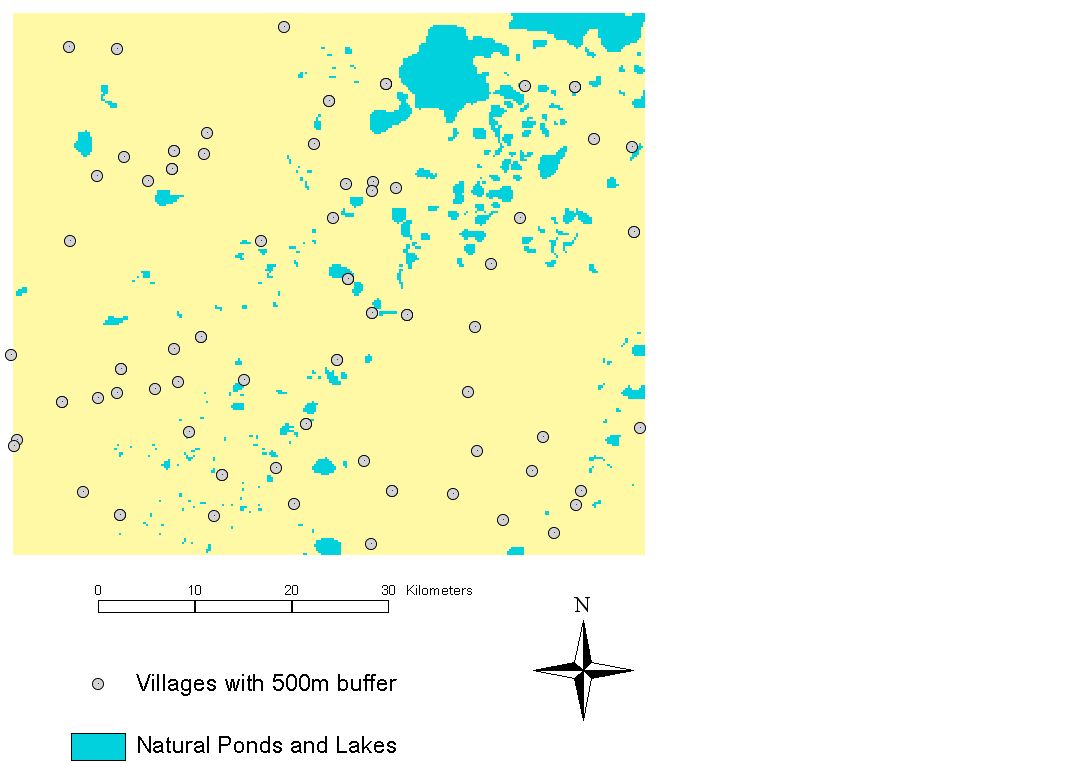


**Figure S2. Typical result of the optimisation process used to determine the thresholds *v* (for NDVI) and *w* (for MNDWI).** This figure shows the result of the optimisation of the threshold w for one 8 day-period (between days 81 to 89). We determined the value of w that minimised both the number of cells identified as flooded vegetation and the mean distance of GPS points to these cells. In this case the value determined for *w* was -0.3. We ran the same process for all 8-day periods and used the minimum value for both thresholds. The final values were *v* = 0.2 and *w* = -0.3.


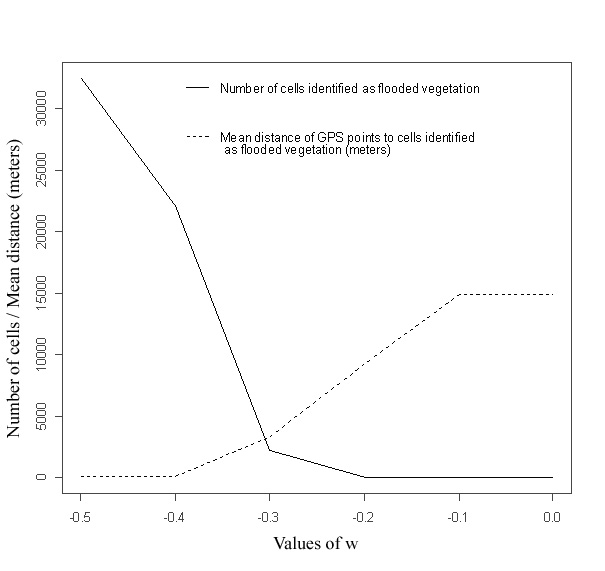

Supplement: Additional file 1 — Construction of the fifth indicator and additional figures. This file explains how was built the fifth environmental indicator used as an explanatory variable in our distribution model. We describe the two steps and the optimisation process leading to the variable called 'distance to flooded vegetation areas suitable for wild birds'. The files also contains figure S1 showing the 64 villages and the natural ponds and lakes included in the study area, and figure S2 showing typical result of the optimisation process used to build the fifth environmental indicator. [file 1476-072X-10-60-S1.DOC]
